# Ideal algorithms in healthcare: Explainable, dynamic, precise, autonomous, fair, and reproducible

**DOI:** 10.1371/journal.pdig.0000006

**Published:** 2022-01-18

**Authors:** Tyler J. Loftus, Patrick J. Tighe, Tezcan Ozrazgat-Baslanti, John P. Davis, Matthew M. Ruppert, Yuanfang Ren, Benjamin Shickel, Rishikesan Kamaleswaran, William R. Hogan, J. Randall Moorman, Gilbert R. Upchurch, Parisa Rashidi, Azra Bihorac

**Affiliations:** 1 Department of Surgery, University of Florida Health, Gainesville, Florida, United States of America; 2 Precision and Intelligent Systems in Medicine (PrismaP), University of Florida, Gainesville, Florida, United States of America; 3 Departments of Anesthesiology, Orthopedics, and Information Systems/Operations Management, University of Florida Health, Gainesville, Florida, United States of America; 4 Department of Medicine, University of Florida Health, Gainesville, Florida, United States of America; 5 Department of Surgery, University of Virginia, Charlottesville, Virginia, United States of America; 6 Department of Biomedical Informatics, Emory University School of Medicine, Atlanta, Georgia, United States of America; 7 Department of Health Outcomes & Biomedical Informatics, College of Medicine, University of Florida, Gainesville, Florida, United States of America; 8 Department of Medicine, University of Virginia, Charlottesville, Virginia, United States of America; 9 Departments of Biomedical Engineering, Computer and Information Science and Engineering, and Electrical and Computer Engineering, University of Florida, Gainesville, Florida, United States of America

## Abstract

Established guidelines describe minimum requirements for reporting algorithms in healthcare; it is equally important to objectify the characteristics of ideal algorithms that confer maximum potential benefits to patients, clinicians, and investigators. We propose a framework for ideal algorithms, including 6 desiderata: explainable (convey the relative importance of features in determining outputs), dynamic (capture temporal changes in physiologic signals and clinical events), precise (use high-resolution, multimodal data and aptly complex architecture), autonomous (learn with minimal supervision and execute without human input), fair (evaluate and mitigate implicit bias and social inequity), and reproducible (validated externally and prospectively and shared with academic communities). We present an ideal algorithms checklist and apply it to highly cited algorithms. Strategies and tools such as the predictive, descriptive, relevant (PDR) framework, the Standard Protocol Items: Recommendations for Interventional Trials-Artificial Intelligence (SPIRIT-AI) extension, sparse regression methods, and minimizing concept drift can help healthcare algorithms achieve these objectives, toward ideal algorithms in healthcare.

## Introduction

The breadth and complexity of human disease confer unique challenges in clinical decision-making. The 10th revision of the International Statistical Classification of Diseases and Related Health Problems (ICD) classification system includes approximately 68,000 diagnostic codes. Patients can have nearly any combination of these diagnoses managed with nearly any combination of relevant therapies whose efficacy hinges on underlying behavioral, social, and genetic determinants of health. Patients and clinicians face shared clinical decision-making tasks while under time constraints and high cognitive loads from high volumes of information [1,2]. The average person generates more than 1 million gigabytes of healthcare data during their lifetime or approximately 300 million books; these massive volumes of data far exceed human cognitive capacities, which allow for approximately 5 to 10 facts per decision [3,4]. Abnormal or unexpected data are typically applied to hypothetical-deductive reasoning and heuristic processes that are highly variable and error prone; when collected data are within normal limits, they are often discarded to reduce cognitive load [5,6]. Unsurprisingly, clinical decision-making errors are common and associated with mortality and morbidity [7,8].

By contrast, high-complexity and high-volume data can be parsed by machine learning applications with relative ease. Published algorithms supporting clinical decisions have become ubiquitous. Hundreds of retrospective studies are classified as artificial intelligence (AI) clinical trials, but few are methodologically rigorous [9,10]. Experts have described important components of algorithm-based and AI-enabled decision support and reporting guidelines; the minimum information about clinical artificial intelligence modeling (MI-CLAIM) checklist, Standard Protocol Items: Recommendations for Interventional Trials-Artificial Intelligence (SPIRIT-AI) extension, and Consolidated Standards of Reporting Trials-Artificial Intelligence (CONSORT-AI) guidelines facilitate consistent reporting, interpretation, and validation of AI applications by establishing minimum requirements [11–15]. We believe that it is equally important to objectify the characteristics of ideal algorithms that confer maximum potential benefits to patients, clinicians, and investigators. To this end, we propose a framework for ideal algorithms consisting of 6 desiderata. This framework is supported by a checklist, which we apply to prominent healthcare algorithms.

## The ideal algorithm framework

**Fig 1.**
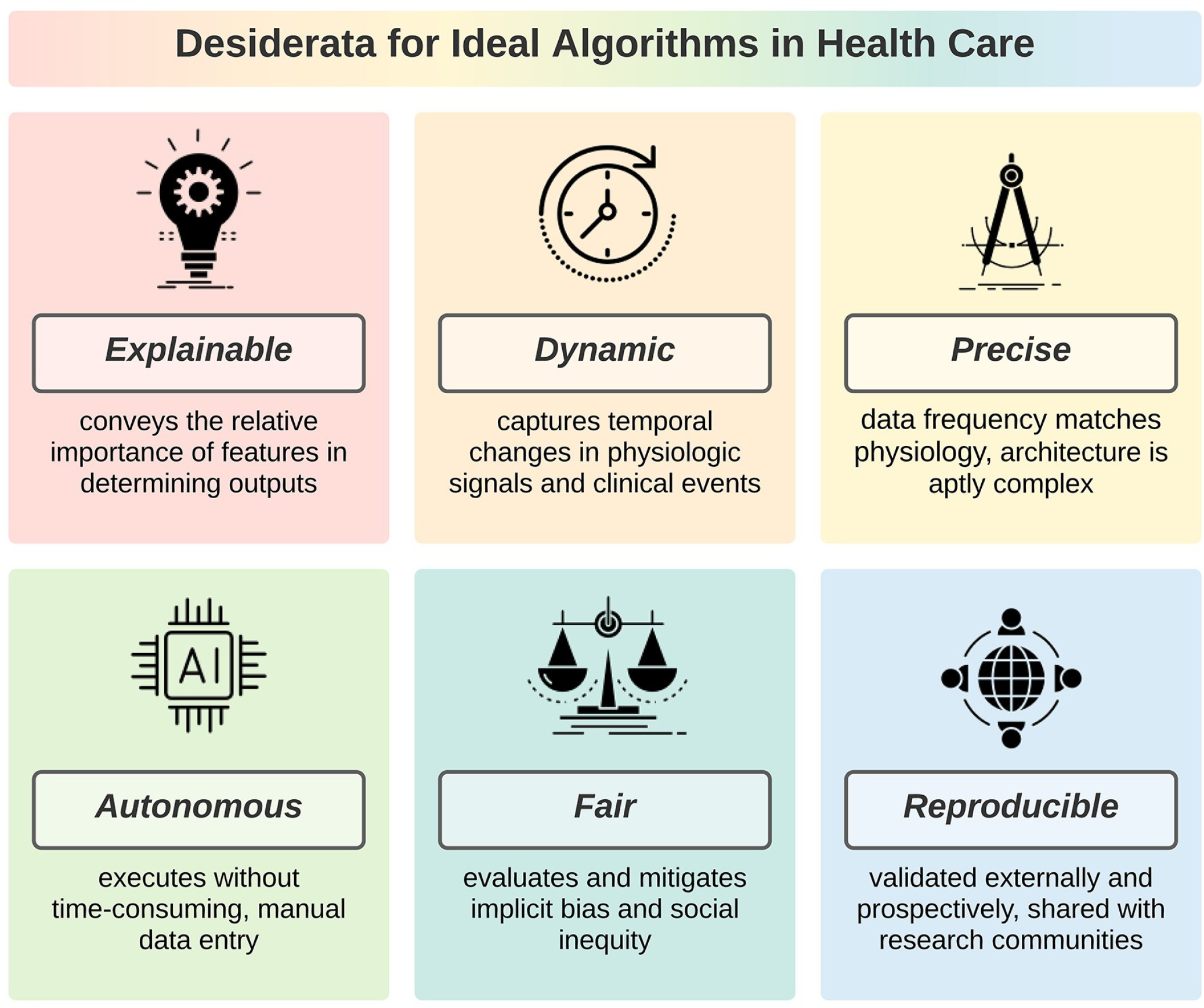
Ideal algorithms in healthcare have 6 desirable characteristics: explainable, dynamic, precise, autonomous, fair, and reproducible.

Ideal algorithms are explainable, dynamic, precise, autonomous, fair, and reproducible, as illustrated in Fig 1. These desiderata are independent. Therefore, the degree to which an algorithm achieves maximum potential benefits to patients, clinicians, and investigators can be conceptualized as a continuum ranging from 0 desiderata (least ideal) to all 6 (most ideal). Desiderata may have unique applications for different algorithms types; our framework is designed to apply broadly to any algorithm type using objective criteria. A checklist of criteria for ideal algorithms is provided in Table 1. Each desideratum is evaluated as being met, partially met, not met, or not applicable by 1 or more objective criteria, which are each evaluated as being met, not met, or not applicable.

## Ideal algorithms are explainable

Explainable algorithms convey the relative importance of features in determining outputs. Informed patients, diligent clinicians, and scrupulous investigators want to know how algorithm predictions are made. We recommend the predictive, descriptive, relevant (PDR) framework for achieving optimal explainability. PDR standardizes discussions regarding machine learning explanations according to predictive accuracy, descriptive accuracy (i.e., the ability of explainability mechanisms to describe objectively what the model has learned), and relevancy as judged by the algorithm’s target human audience for its ability to provide insight into a chosen problem [16].

Algorithm predictive accuracy is commonly described and easily interpreted by most clinicians and scientists. Yet, one underappreciated aspect of predictive accuracy can affect the explainability of model outputs: In some cases, prediction error varies substantially by class. When applying an algorithm to a patient in a class with disproportionately high prediction error, one should have less confidence that model outputs are accurate and deemphasize algorithm outputs in the decision-making process. Descriptive accuracy, or objective indicators of what the model learned (e.g., coefficients in a regression model or weights in a neural network), is less commonly described and is difficult to achieve with complex, “black box” models such as deep neural networks. By contrast, the odds ratios produced by simple logistic regression are relatively easy to interpret, allowing clinicians to understand and mentally simulate the model’s process for generating predictions. Despite the greater descriptive accuracy of simple models, complex models are often needed to solve complex, nonlinear problems for which simple models suffer from poor predictive accuracy. Therefore, algorithm explainability methods have focused on complex machine and deep learning models. We note, though, recent studies showing no great superiority of deep learning over regression in this field of classifying illness severity of individual patients using readily available clinical data [15,17]. Descriptive accuracy can be improved by choosing a simple, highly explainable model or performing post hoc analyses on a trained, complex model to understand what relationships the model has learned [18]. Finally, the PDR framework holds that relevancy is context specific, i.e., the usefulness of model explainability mechanisms depends on criteria that are unique to different people groups. Therefore, relevancy should be graded by the intended human audience and their intended use of predictions generated by the model.

Examination of relevancy can resolve trade-offs between predictive and descriptive accuracy [16]. Consider an algorithm that is predicting the risk for complications after surgery. To target researchers who seek the greatest predictive accuracy, explainability mechanisms could be used to optimize feature engineering. To target patients who are planning to undergo elective surgery, explainability mechanisms could be used to identify the most important modifiable risk factors for complications (i.e., modifiable predictors of wound infection could include poor blood glucose control and ongoing tobacco use). Notably, the PDR framework (intentionally) does not address causal inference or methods for determining the degree to which altering one variable changes another. In its purest form, explainability describes general relationships and does not distinguish between causal and noncausal effects. Therefore, PDR is a simple and effective framework for evaluating and discussing the full range of user-specific machine learning interpretations without confusing explainability with causality.

## Ideal algorithms are dynamic

Dynamic algorithms capture temporal changes in physiologic signals and clinical events via time series or sequence modeling. When algorithms are intended to improve clinical trial design, statistical adjustment, or patient enrollment strategies, static predictions at a single time point are adequate. When algorithms are intended to augment real-time, clinical decision-making as conditions evolve, the algorithm should make dynamic predictions using new data as it become available. Dynamic algorithm predictions are useful because continuous manual recalculations are burdensome for individual patients, caregivers, and clinicians, and the cognitive load imposed by serial reassessments of continuously accumulating data is substantial. Potentially valuable information is easily missed and underutilized for risk stratification and clinical decision-making, as it often requires computational capacity beyond human ability [1,2]. Instead, humans tend to rely on heuristics, or cognitive shortcuts, which can lead to bias, error, and preventable harm [6,19]. By contrast, large volume electronic health record (EHR) data are well suited to dynamic predictive analytics that capture trends over time; physiologic time series data have been used to predict mortality and specific conditions such as acute kidney injury [20–23].

Algorithm dynamicity is especially important when modeling conditions that change rapidly. For instance, intracranial and cerebral perfusion pressure can vacillate substantially after traumatic brain injury. Delayed recognition of rapid changes in intracranial and cerebral perfusion pressure can worsen outcomes because brain ischemia is exquisitely time sensitive. Classical traumatic brain injury prediction models only used static variables present on admission [24–26]. These models may be useful for research purposes, early prognostication, and early resource use decisions, but they do not perform the critically important function of updating predictions as new data become available. For example, an algorithm using 5-minute median values of intracranial pressure, mean arterial pressure, cerebral perfusion pressure, and Glasgow coma scale scores predicts 30-day mortality with approximately 84% discrimination 48 hours after admission [25]. Using 5-minute median values rather than continuous data streams may be favorable for implementation in clinical settings, where data collection is frequently interrupted.

Dynamic algorithms face challenges in evaluating performance over time and explainability. In some cases, algorithms learn to predict which action a clinician will take next, rather than physiologic events [27,28]. In addition, there are no standards for evaluating model performance when predictions are made in a continuous or nearly continuous fashion. We suggest evaluating standard model performance metrics at several predetermined, discrete time points, including the point at which enough information has become available that calibration is expected to plateau, achieving continuous monitoring of predictive performance. To optimize explainability for dynamic algorithms, attention mechanisms can reveal periods during which certain features make significant contributions to algorithm outputs [20,29]. For example, the DeepSOFA algorithm uses time series measurements of the same input variables as the sequential organ failure assessment (SOFA) score, passing those values through a recurrent neural network with gated return and self-attention units. In 2 independent datasets of intensive care unit (ICU) patients, DeepSOFA predicted in-hospital mortality with accuracy greater than that of the traditional SOFA score [20]. Model explainability was promoted by generating heatmaps that illustrate each variable’s relative contributions at each time step to the model’s ultimate mortality prediction. Using time series measurements in dynamic algorithms relates to the next desideratum of ideal algorithms: precision.

## Ideal algorithms are precise

Precise algorithms use data collection rates that are proportional to rates of physiologic changes and machine learning techniques whose complexity matches the target outcome. Precision is important because human diseases are complex and nonlinear [30,31]. Simple, additive models often demonstrate poor predictive performance [32–34]. Three days after colorectal surgery, a serum C-reactive protein level less than 172 mg/L has a 97% negative predictive value for the occurrence of anastomotic leak [34]. This finding may facilitate early discharge home after major surgery. However, high C-reactive protein levels are nonspecific: As a general marker of systemic inflammation, one would expect that C-reactive protein has a poor positive predictive value, and it does (21%). To perform a complex task, such as differentiating between an anastomotic leak and other pro-inflammatory postoperative complications, it is potentially advantageous to incorporate high-resolution, multimodal patient data and machine learning modeling [35–39].

For a given algorithm, the ideal rate of data collection should exceed by several fold the rate of salient physiologic changes, similar to the manner in which Harry Nyquist noted that to represent a signal with fidelity, sampling should occur at twice the highest frequency of the signal [40]. In many disease processes, this will require high-resolution data that are sampled at a frequency that allows for early diagnosis, prevention, or treatment by capturing subtle but clinically significant physiologic changes. Generally, longer intervals are more likely to miss critical physiologic changes that occur between measurements [41–44]. For hospitalized patients, high-frequency assessments are associated with greater accuracy in predicting decompensation. Subtle signs of physiologic instability often occur hours before organ failure and cardiac arrest, representing opportunities for prevention [45,46]. This is discordant with standard practices on hospital wards, where vital signs are typically measured every 4 hours. Unsurprisingly, continuous vital sign monitoring is associated with fewer rescue events, respiratory decompensation events, unplanned ICU transfers, and ICU days, as well as shorter hospital length of stay [47,48]. Yet, continuous monitoring can be expensive, can generate distracting false-positive alarms, might impair patient comfort and mobility, and is not supported by a great deal of level 1 evidence, apart from heart rate characteristics monitoring for neonatal sepsis [49–54]. Therefore, continuous monitoring is often reserved for high-risk patients that are most likely to manifest time-sensitive clinically significant physiologic changes, for whom continuous data have a proven ability to stand alone [55–60] and to add information to EHR data elements [36,59,61–64] in predictive analytics. In designing algorithms, we suggest resampling data at intervals that align with the expected velocity with which changes in physiologic signals lead to clinically significant events, with sampling frequency equal to or greater than the Nyquist rate [40].

In many healthcare settings, highly granular data are routinely recorded from multiple sources for clinical purposes. For example, clinical surveillance of critically ill patients often includes not only vital sign and laboratory measurements but also assessments of mental status, pain, respiratory mechanics, and mobility. Historically, these assessments are performed and recorded by hospital staff in a subjective fashion. With improvements in sensor technologies and machine learning applications in healthcare, it has become feasible to automatically capture and analyze data from ICU patients and environments tracked by accelerometers, light sensors, sound sensors, and high-resolution cameras [65]. Wearable sensors can also capture meaningful, multimodal physiologic data from community-dwelling participants. Notably, high-resolution, multimodal data often suffer from high dimensionality, rendering simple algorithms inaccurate.

Conversely, when algorithms have too many inputs relative to their application, generalizability is compromised due to overfitting. The optimal approach balances predictive accuracy and input complexity by using the fewest variables necessary to maintain high performance. This can be accomplished with sparse regression methods [66]. Generating parsimonious models, although harboring the potential to compromise predictive performance, has the additional advantage of improving the descriptive accuracy for input features, as described above in the “Ideal algorithms are explainable” section.

## Ideal algorithms are autonomous

Autonomous algorithms execute with minimal human input. Beyond the training and testing autonomy shared by all unsupervised machine learning algorithms, autonomous algorithms in healthcare can be implemented with minimal input by users. Manual data entry by the end user imposes time constraints that hinder the clinical application of nonautonomous decision support algorithms [67]. For dynamic models that capture temporal changes by frequently resampling high-resolution data, the cost of manual data entry is even greater. Fortunately, the widespread availability of high-volume EHR data and open-source machine learning code promotes algorithm autonomy [68,69].

Autonomous algorithms have substantial potential to augment decision-making for clinical scenarios in which many input features have complex associations with outcomes. Predicting risk for complications after surgery is one such instance. Accurate predictions of postoperative complications can influence patients’ decisions whether to undergo surgery, identify risk factors that are amenable to risk reduction strategies, and inform decisions regarding appropriate postoperative triage destination and resource use. Regrettably, clinicians demonstrate variable performance in predicting risk for postoperative complications, and surgeons frequently commit judgment errors that confer preventable harm [70–72]. Several accurate predictive analytic decision support algorithms have been developed and validated to augment clinical risk predictions, but most are hindered by time-consuming manual data entry requirements and lack of integration with clinical workflow [67,73–77]. Yet, autonomous prediction of postoperative complications is possible. One machine learning platform autonomously imports EHR input data to predict 8 postoperative complications with area under the receiver operating characteristic curve (AUC) 0.82 to 0.94, exhibiting accuracy greater than that of physicians [69,70].

Potential advantages of autonomy also apply to algorithm training. Supervised machine learning algorithms use training data that are labeled by humans and then classifies or makes predictions on new, unseen data; in unsupervised learning, algorithms generate their own labels according to the structure and distribution of input data, discovering patterns and associations. Deep learning models avoid time-intensive, handcrafted feature engineering by autonomously learning feature representations from raw data. In addition to efficiency and pragmatism, autonomous learning offers performance advantages, as has been demonstrated in the gaming industry. “Go” has 32,490 possible first moves, precluding an exhaustive search of all possible moves for each board configuration. Instead, a combination of deep and reinforcement learning can predict outcomes following sequences of actions and efficiently identify optimal moves. This approach was initially applied in learning 30 million positions and instructions from a human Go expert, allowing the algorithm to build a decision policy network. The program then played against itself, attempting to maximize the chance of beating previous versions of its own decision-making policy. Next, a value network predicted the final outcome of a game based on any board configuration. Finally, the policy and value networks were combined, and an optimized search algorithm was used to select the next move for any board configuration. This approach defeated the European Go champion 5 games to 0 [78]. Subsequently, a completely autonomous model was trained exclusively on self-play. This model defeated the human input model 100 games to 0 [79]. For healthcare applications, it remains plausible that performance is greatest for completely autonomous learning approaches for instances in which high-quality training data exist. Unfortunately, most healthcare data sources are compromised by a lack of granularity, generalizability, volume, or a combination thereof.

## Ideal algorithms are fair

Fair algorithms evaluate and mitigate implicit bias and social inequity. In theory, algorithms use mathematical formulas and functions to produce objective outputs, offering a bulwark against subjectivity with resultant bias and inequity. In practice, many algorithms are trained on biased source data and produce biased outputs [80]. In healthcare, single-center source data may disproportionately represent certain demographics. When these data are used for algorithm training, that algorithm may perform poorly when applied to a patient that is sparsely represented in the source data. Poor performance may be especially harmful when it has directionality, i.e., the algorithm consistently overestimates or underestimates risk in a manner that affects decision-making. For example, if a decision support tool incorporates the observation that Black patients have increased risk for mortality after coronary artery bypass, then model outputs could decrease the likelihood that Black patients will garner the benefits of an indicated procedure [81,82]. To determine whether a demographic or socioeconomic factor should be included in a prediction model, it is necessary to assess whether that factor has a plausible or proven pathophysiologic association with the outcome of interest. To do so, we recommend machine learning explainability mechanisms, causal inference, and clinical interpretation of biologic plausibility. If this analysis reveals no evidence of a pathophysiologic association, then it is likely that the demographic or socioeconomic factor is an indicator of suboptimal access to care, referral patterns, or systemic bias and should be excluded from the algorithm.

Algorithm bias can be evaluated by assessing calibration across demographic and socioeconomic variables. If an observed outcome matches algorithm-predicted probabilities for men but not women, then the algorithm exhibits bias against women. This method was used to evaluate racial bias in an algorithm that predicts healthcare needs [83]. The authors compared observed versus predicted healthcare needs for primary care patients who self-identified as Black versus White. When comparing Black and White patients with similar predicted risk, Black patients had greater illness severity. The algorithm was designed to identify patients at or above the 97th percentile of risk and allocate them to receive extra care. At the 97th percentile, Black patients had 4.8 chronic illnesses, and White patients had 3.8 chronic illnesses (*p* < 0.001). The likely mechanism for this discrepancy was the use of healthcare expenditures as a proxy for health needs. If less money is spent on Black patients than on White patients who have the same illness severity, then the algorithm will errantly learn that Black patients have lesser health needs than White patients who have the same illness severity. Racial discrepancies were eliminated by modifying the algorithm so that expenditures were not a proxy for health needs.

Several other methods for promoting algorithm fairness have been described [4]. Models should be reevaluated over time to determine whether temporal changes in study populations, healthcare systems, and medical practices have affected relationships between features and outcomes. This phenomenon, concept drift, undermines algorithm performance by several mechanisms, including algorithm bias. During preprocessing, individual patient data can be mapped to probability distributions that obfuscates information about membership in a protected subgroup (e.g., race, ethnicity, sex, gender, etc.) while retaining as much other information about the patient as possible [84]. During postprocessing, the open-source What-If Tool allows interactive model testing under user-controlled hypothetical circumstances, which can quantify the effects of different demographic and socioeconomic factors on model outputs [85]. In addition, the What-If Tool can demonstrate whether model performance varies across subgroups, which may be useful in determining whether the model should be applied for a patient that is poorly represented in model training data.

## Ideal algorithms are reproducible

Reproducible algorithms are validated both externally and prospectively and are shared with academic communities. In a survey distributed by *Nature*, greater than 70% of all researchers had attempted and failed to reproduce another scientist’s experiments, and 90% reported that science is facing a reproducibility crisis [86]. Reproducibility, a critically important element of any scientific inquiry, is especially important for machine learning algorithms because it establishes trustworthiness and credibility. Prior to successful clinical implementation, “black box” algorithms must earn the trust of patients, clinicians, and investigators. Even when explainability is suboptimal, people may be willing to use an algorithm that is well validated and freely available to academicians. In addition, a reproducible algorithm can be tuned and optimized over time, offering a performance advantage.

There are several major barriers to algorithm reproducibility. Prominent EHR platforms are not designed to accommodate algorithm scalability across institutions and platforms. This produces an “analytic bottleneck” in which investigators must process, harmonize, and validate massive amounts of data within institutional silos. Many researchers do not possess the necessary resources to work at such a large computational scale, much less keep track of which data were used for different studies and evaluate the impact of data reuse on the statistical bias. In addition, there are limited cloud resources for sharing multiple, large, healthcare data repositories among research groups that have their own algorithm pipelines and tools. Given these obstacles, many algorithms are never shared and validated externally. To ensure that algorithms are suitable for external validation, results from interventions using AI algorithms should be reported in a standardized fashion, as proposed by the SPIRIT-AI extension, which was developed in parallel with CONSORT-AI guidelines [11,12]. Compliance with these protocols will promote the reproducibility of findings. Yet, most reports involving algorithms in healthcare do not involve implementation in a clinical trial. Noninterventional studies that involve prediction models should comply with the Transparent Reporting of a Multivariable Prediction Model for Individual Prognosis or Diagnosis (TRIPOD) statement [87,88]. Finally, generalizability can be enhanced by using input features that are collected routinely in clinical care, excluding features whose collection requires specialized measurement tools that are unavailable in most settings.

Federated learning offers opportunities to ensure the external validity, generalizability, and reproducibility of algorithms via collaborative machine learning without data sharing [89–92]. When sensitive patient data are shared between institutions, there is risk for unintended data disclosures and piracy by adversarial third parties. In federated learning, local models are trained separately and consolidated into a global model [89–92]. As local models train, they send local updates in the form of gradients or coefficients for incorporation in the global model. Even when these relatively secure methods are applied, privacy leakage can occur when adversaries infer whether a given attribute belongs to the model’s training data or infer class representatives from collaborative models [93–97]. To mitigate privacy leakage in federated learning, the risks for privacy-sensitive information and privacy leakage can be quantified for each data record, with subsequent obfuscation of high-risk records.

## Application of the ideal algorithms framework to prominent algorithms in healthcare

To identify prominent examples of algorithms in healthcare, we reviewed the 20 most highly cited articles in medical AI, as identified in a bibliometric analysis by Nadri and colleagues [98]. Among these 20 articles, 8 described an algorithm. Table 2 applies the ideal algorithm framework to these 8 algorithms by the majority vote of 3 independent raters. Fleiss kappa statistic was 0.708, suggesting substantial interrater agreement [99,100]. All 8 algorithms met criteria for precision, 6 of the algorithms were autonomous, 5 were fair, 4 were explainable, and 3 were reproducible. Dynamicity was not applicable to any of the algorithms. These findings suggest opportunities to enhance the autonomy, fairness, explainability, and reproducibility of algorithms in healthcare.

## Conclusions

While the breadth and complexity of human disease compromise the efficacy of hypothetical-deductive reasoning and heuristic decision-making, high-complexity and high-volume data can be parsed by machine learning applications with relative ease. Established guidelines describe minimum requirements for reporting algorithm healthcare applications; it is equally important to describe the maximum potential of ideal algorithms. We propose that ideal algorithms have 6 desiderata that are represented in a checklist presented herein: explainable (convey the relative importance of features in determining outputs), dynamic (capture temporal changes in physiologic signals and clinical events), precise (use high-resolution, multimodal data and aptly complex architecture), autonomous (learn with minimal supervision and execute without human input), fair (evaluate and mitigate implicit bias and social inequity), and reproducible (are validated externally and prospectively and shared with academic communities). By achieving these objectives, healthcare algorithms confer maximum potential benefits to patients, clinicians, and investigators.

## Figures and Tables

**Table 1.** Checklist for ideal algorithms in healthcare.

Desiderata	Criteria	Yes	Location	No	N/A
**Explainable** Yes^a^ Partially^b^ No^c^ N/A^d^	*Feature importance:* conveys the relative importance of features in determining algorithm outputs				
	*Descriptive accuracy:* describes what the algorithm has learned (e.g., weights in a neural network)				
	*Simulatability:* clinicians can understand and mentally simulate the model’s process for generating predictions				
	*Relevance:* describes relevancy as judged by the algorithm’s target human audience				
**Dynamic** Yes^a^ Partially^b^ No^c^ N/A^d^	*Temporality:* captures temporal changes in physiologic signals and clinical events				
	*Continuous monitoring:* performance is reassessed at several time points, including the point at which performance is expected to plateau				
**Precise** Yes^a^ Partially^b^ No^c^ N/A^d^	*Data frequency:* rate of data collection matches the rate of physiologic changes				
	*Complexity:* algorithm complexity matches the complexity of the prediction or classification task				
**Autonomous** Yes^a^ Partially^b^ No^c^ N/A^d^	*Efficiency:* the algorithm executes without the need for time-consuming, manual data entry by the end user (i.e., patient, provider, or investigator)				
**Fair** Yes^a^ Partially^b^ No^c^ N/A^d^	*Generalizability:* algorithm is developed and validated across diverse patient demographics and practice settings				
	*Selectivity*: excludes features that lack pathophysiologic or linguistic association with outcomes, but may introduce bias				
	*Objectivity:* includes variables that are minimally influenced by clinician judgments (e.g., vital signs)				
**Reproducible** Yes^a^ Partially^b^ No^c^ N/A^d^	*Generalizability*: validated externally, prospectively				
	*Collaboration*: algorithm is shared with the research community				
	*Compliance:* fulfills SPIRIT-AI extension guidelines (if trial) and fulfills CONSORT-AI guidelines				

a Overall adjudication is “Yes” when all criteria are either met or not applicable.

b Overall adjudication is “Partially” when some but not all criteria are either met or not applicable.

c Overall adjudication is “No” when no criteria are met.

d Overall adjudication is “N/A” when all criteria are not applicable.

CONSORT-AI, Consolidated Standards of Reporting Trials-Artificial Intelligence; N/A, not applicable; SPIRIT-AI, Standard Protocol Items: Recommendations for Interventional Trials-Artificial Intelligence.

**Table 2.** Highly cited AI algorithms graded by their interpretability, dynamicity, precision, autonomy, fairness, and reproducibility.

Primary author	Algorithm application	Explainable	Dynamic	Precise	Autonomous	Fair	Reproducible
Gulshan	Detecting diabetic retinopathy	No	N/A	Yes	Yes	Yes	No
Iorio	Predicting tumor sensitivity to pharmacotherapies	Yes	N/A	Yes	No	Yes	No
Kamnitsas	Brain lesion segmentation	Yes	N/A	Yes	Yes	Yes	Yes
Ott	Predicting human lymphocyte antigen binding	No	N/A	Yes	Yes	Yes	Yes
Savova	Extracting information from clinical free text in EHRs	No	N/A	Yes	Yes	No	Yes
Tajbakhsh	Medical image classification, detection, and segmentation	No	N/A	Yes	Yes	Yes	No
Wolfe	Identifying and assessing severity of fibromyalgia	Yes	N/A	Yes	No	No	No
Xiong	Predicting splicing regulation for mRNA sequences	Yes	N/A	Yes	Yes	No	No

AI, artificial intelligence; EHR, electronic health record; N/A, not applicable (i.e., temporal changes and continuous monitoring were not applicable to these algorithms and their intended use).
